# STRIDE: a command-line HMM-based identifier and sub-classifier of *Plasmodium falciparum* RIFIN and STEVOR variant surface antigen families

**DOI:** 10.1186/s12859-021-04515-8

**Published:** 2022-01-06

**Authors:** Albert E. Zhou, Zalak V. Shah, Katie R. Bradwell, James B. Munro, Andrea A. Berry, David Serre, Shannon Takala-Harrison, Timothy D. O’Connor, Joana C. Silva, Mark A. Travassos

**Affiliations:** 1 Malaria Research Program, Center for Vaccine Development and Global Health, University of Maryland School of Medicine, Baltimore, MD USA; 2 Institute for Genome Sciences, University of Maryland School of Medicine, Baltimore, MD USA; 3 Program in Personalized and Genomic Medicine, University of Maryland School of Medicine, Baltimore, MD USA; 4 Department of Microbiology and Immunology, University of Maryland School of Medicine, Baltimore, MD USA

**Keywords:** Malaria, *Plasmodium falciparum*, RIFIN, STEVOR, Bioinformatics, Hidden Markov models

## Abstract

**Background:**

RIFINs and STEVORs are variant surface antigens expressed by *P. falciparum* that play roles in severe malaria pathogenesis and immune evasion*.* These two highly diverse multigene families feature multiple paralogs, making their classification challenging using traditional bioinformatic methods.

**Results:**

STRIDE (STevor and RIfin iDEntifier) is an HMM-based, command-line program that automates the identification and classification of RIFIN and STEVOR protein sequences in the malaria parasite *Plasmodium falciparum*. STRIDE is more sensitive in detecting RIFINs and STEVORs than available PFAM and TIGRFAM tools and reports RIFIN subtypes and the number of sequences with a FHEYDER amino acid motif, which has been associated with severe malaria pathogenesis.

**Conclusions:**

STRIDE will be beneficial to malaria research groups analyzing genome sequences and transcripts of clinical field isolates, providing insight into parasite biology and virulence.

**Supplementary Information:**

The online version contains supplementary material available at 10.1186/s12859-021-04515-8.

## Background

**Fig. 1:**
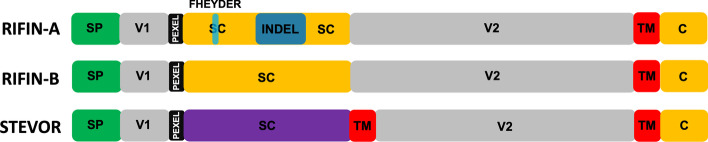
General structure of RIFINs and STEVORs. RIFINs and STEVORs are expressed on the surface of an erythrocyte infected with *P. falciparum*. Protein domains are illustrated as green (signal peptide), grey (variable domains), red (transmembrane domains), blue (25 amino acid insertion), and orange and purple (semi-conserved domains). There are approximately 160 *rif* genes in the 3D7 reference genome, separated into two subtypes, RIFIN-A and RIFIN-B, depending on sequence and subcellular localization. The FHEYDER motif (in blue) is present in the semi-conserved domain of 36 RIFIN-As in the 3D7 reference strain. STEVORs encompass ~ 30 genes per genome and are structurally similar to RIFIN-Bs

The eukaryotic parasite *Plasmodium falciparum* features the repetitive interspersed family (RIFIN) and subtelomeric variable open reading frame (STEVOR) multigene family, variant surface antigens that are associated with severe malaria pathogenesis and immune evasion [1–3]. RIFINs and STEVORs share a domain architecture, although RIFINs can be further subtyped into RIFIN-As and -Bs based on a 25 amino acid indel in the semi-conserved domain and differences in subcellular localization suggestive of distinct functions (Fig. 1) [4, 5]. A subset of RIFIN-As harboring a seven amino acid FHEYDER motif in the semi-conserved domain have been shown to inhibit B- and NK-cell activation, weakening host defenses against malaria infection [6]. Both protein families are also targets of natural immunity [7].

RIFINs and STEVORs pose challenges in genomic analyses due to their immense genetic diversity and numerous paralogs, which cause difficulties in reference-based assembly and identification. There are limited bioinformatic approaches to distinguish between RIFINs and STEVORs and to further classify RIFINs to the subtype level. Apart from laborious sequence alignment and phylogenetic analyses, BLAST is one of the few available tools [8]. However, BLAST requires a comprehensive reference index, lacks the sensitivity to detect highly divergent sequences, and cannot readily delineate between RIFIN subtypes. In contrast, profile Hidden Markov Models (HMM) offer not only better accuracy and speed, but also sensitivity in detecting remote homologs [9]. Three HMM-based tools have been used to categorize RIFIN and STEVOR sequences: RSpred [4], TIGRFAM [10], and PFAM [11]; however, each is built using limited sets of reference RIFIN and/or STEVOR sequences. The more recent tools TIGRFAM and PFAM, as part of the Interpro database [11], do not subtype RIFINs or automatically assign annotations. While RSpred addressed these concerns, it was web-based, could only evaluate ten sequences per job, and its web interface is no longer responsive.

Here, we introduce an improved HMM-based, command-line program called STRIDE (STevor and RIfin iDEntifier). STRIDE has better sensitivity than available HMM tools to detect both RIFINs and STEVORs, and also features RIFIN subtyping, automated annotations, and adjustable thresholds for sensitivity and specificity. Importantly, STRIDE allows for the determination of the number of RIFIN-A sequences with a FHEYDER motif, providing insight into mechanisms to weaken host defenses. STRIDE will have particular value for malaria genomic epidemiologists, as next-generation sequencing of clinical field isolates increases in prevalence and the contributions of RIFIN and STEVOR multigene families to severe malaria pathogenesis and the acquisition of natural immunity to malaria become clearer.

## Implementation

STRIDE consists of a merged HMM generated from three different refined multiple sequence alignments of full-length publicly available RIFIN and STEVOR protein sequences (Additional file 1: Figs. S1 and S2). A total of 3536 RIFIN and STEVOR sequences were downloaded from PlasmoDB (Release 45; August 28, 2019, keyword: “RIFIN/STEVOR”). Redundant sequences were clustered with CD-HIT v4.6 (option*:* -c 1.0). RIFIN-A, RIFIN-B, and STEVOR proteins were first identified via BLAST. For each set of protein sequences, a multiple sequence alignment was created, and a corresponding HMM was generated with hmmbuild (default parameters) as part of the HMMER3 v3.2.1 package. In an iterative process (Additional file 1: Fig. S1), we used each HMM profile to search for homologous sequences in other datasets. Sequences with the highest scores were incorporated into a new seed alignment, where another respective HMM profile was created. Training concluded for each HMM profile when no additional sequences could be extracted.

STRIDE uses a FASTA file as input and scores the query sequences against the HMM profile. A subprogram written in Perl v5.24 parses these scores and outputs the sequence classifications as a tab-delimited text file (Additional file 2). The main classifications are “RIFIN-A”, “RIFIN-B”, and “STEVOR.” STRIDE outputs the number of RIFIN-As with a FHEYDER amino acid motif as an exact pattern match. Truncated or highly divergent sequences are designated as “likely” RIFIN or STEVOR, and those that are unable to meet RIFIN subtyping criteria due to insufficient discriminatory characteristics (*e.g.* missing the protein segment containing the defining 25 amino acid indel) are called simply “RIFIN.”

To determine sensitivities and specificities, we created a “validation” dataset that spanned a range of variant surface antigen sequence sizes, including 3888 presumed RIFINs and STEVORs from sequenced clinical isolates and publicly available assemblies (Table 1, Additional file 1: Fig. S2) [12]. In addition, we downloaded annotated protein FASTA files from several *Plasmodium* reference genomes: *P. falciparum* 3D7 (5548 sequences), *P. vivax* (6667 sequences), *P. berghei* strain ANKA (5076 sequences), *P. reichenowi* (5644 sequences), and *P. chabaudi* (5217 sequences) to test our profiles for false positives and negatives.

**Table 1:** Comparison of STRIDE to PFAM and TIGRFAM, using the same parameter values

Dataset	STRIDE								PFAM			TIGRFAM		
	**∑**	**∑** RIFIN	RIFIN-A	RIFIN-B	RIFIN	Likely RIFIN	# FHEYDER	STEVOR	**∑**	RIFIN	STEVOR	**∑**	RIFIN	STEVOR
*P. falciparum 3D7* (185 *rif* + 43 *stevor* = 228)	**220**	182	122	52	4	4	36	38	**216**	178	38	**215**	177	38
*P. reichenowi* (463 *rif* + 66 *stevor* = 529)	**514**	452	250	158	24	20	59	62	**477**	416	61	**498**	436	62
Validation Dataset (3888 sequences)	**3540**	2844	1409	741	318	376	605	696	**2707**	2014	693	**3394**	2694	700
*P. vivax*, *P. berghei*, *P. chabaudi*	**0**	0	0	0	0	0	–	0	**0**	0	0	**0**	0	0

∑ represents the total number of sequences. All three tools had 100% specificity

## Results

### Generation of HMM profiles

**Fig. 2:**
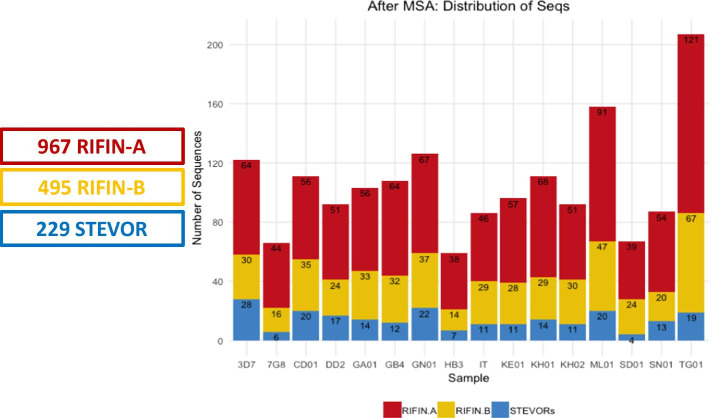
Stacked bar graphs of the sequence distribution from all available *P. falciparum* genomes from PlasmoDB v45 at the conclusion of training each HMM profile. A total of 3536 RIFIN and STEVOR sequences were downloaded from PlasmoDB (Release 45; August 28, 2019). Redundant sequences were clustered with CD-HIT v4.6. HMM (Hidden Markov Model) profiles specific for RIFIN-A, RIFIN-B, and STEVOR proteins were created and iteratively trained against subsets of sequences that were not present in the initial seeding. 967 RIFIN-A, 495 RIFIN-B, and 229 STEVOR sequences comprised the final datasets, providing representation of sequences from all genomes. The Malian (ML01) and Togo (TG01) strains were polyclonal and had overall higher numbers of representative sequences. Of the total of 228 RIFINs and STEVORs annotated in the 3D7 reference genome, STRIDE used 122 3D7 sequences

From the 3536 RIFIN and STEVOR sequences downloaded from PlasmoDB, 967 RIFIN-A, 495 RIFIN-B, and 229 STEVOR sequences comprised the final datasets at the conclusion of HMM training (Fig. 2, Additional file 1: Fig. S2). This included representation of sequences from all sampled genomes. The Malian (ML01) and Togo (TG01) strains were polyclonal and had higher overall numbers of representative sequences. Of the 228 total RIFINs and STEVORs annotated in the 3D7 reference genome, STRIDE incorporated 122 of these sequences.

### Performance evaluation

**Fig. 3:**
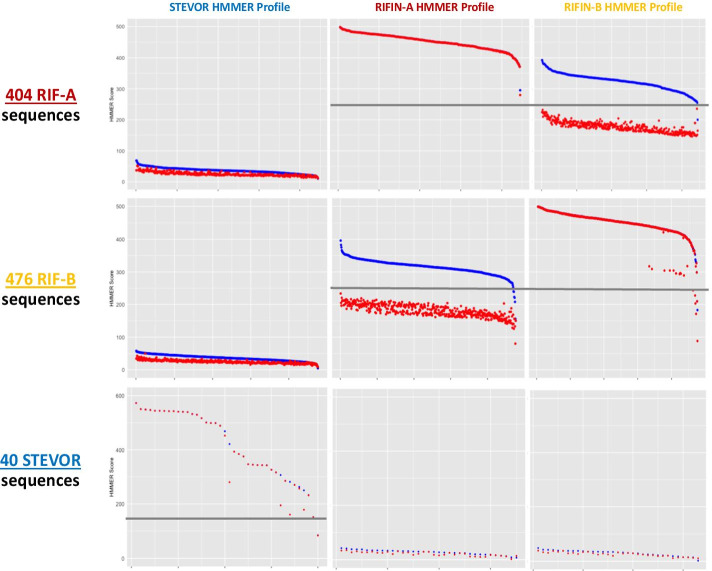
Depicting relationships of HMM Scores. Whole sequence scores are represented in blue and HMM domain scores are represented in red. Sets of sequences excluded from the creation of each HMM profile were used to define the limits of detection, represented by a grey line. Datasets of 404 RIFIN-A, 476 RIFIN-B, and 40 STEVOR sequences excluded from the HMM training were used to test and define the limits of detection for each profile. All RIFIN-A and -B sequences had low concordance to the STEVOR profile, failing to meet its threshold score of 145. The 404 RIFIN-A sequences had whole sequence (blue) and domain (red) scores that exceeded the thresholds for the RIFIN-A profile. In contrast, none of the 404 RIFIN-A sequences met classification criteria for RIFIN-Bs, as their domain scores (red) were below the threshold score of 250. The Y-axis represents HMM scores, and the X-axis represents the ordered numerical label for each sequence

The sensitivity and specificity of STRIDE is adjustable, although default parameters have been optimized to produce the most conservative designations (Fig. 3, Additional file 2). Datasets of 404 RIFIN-A, 476 RIFIN-B, and 40 STEVOR sequences that were randomly selected and excluded from the HMM training were used to test and define the limits of detection for each profile (Fig. 3, Additional file 1: Figs. S1 and S2). All RIFIN-A and -B sequences had low concordance to the STEVOR profile, failing to meet the STEVOR threshold score of 145. The 404 RIFIN-A sequences had whole sequence (represented in blue) and domain (represented in red) scores that exceeded the thresholds for the RIFIN-A profile. In contrast, none of the 404 RIFIN-A sequences met classification criteria for RIFIN-Bs, as their domain scores (red) were below the threshold score of 250. In the same manner, none of the 476 RIFIN-B sequences met the 250 domain threshold score (red) to be classified as a RIFIN-A profile. A set of positive control sequences from 3D7 demonstrated high concordance to each profile illustrated by their respective Circos plot (Additional file 1: Fig. S3).

Based on these findings, we developed an algorithm to specify the type and subtype of a queried sequence based on whole sequence and domain scores (Additional file 2). The first limit of detection determines which of the three profiles (RIFIN-A, RIFIN-B, or STEVOR) registered the greatest whole sequence score. For a queried sequence to be considered a RIFIN, the whole sequence score must surpass a threshold of 200 against either the RIFIN-A or RIFIN-B profile. Queries with whole sequence scores between 100 and 200 to a RIFIN profile are considered “likely RIFINs” and scores ≤ 100 are considered “unlikely RIFINs”. RIFIN subtyping requires a domain score ≥ 250 to a respective RIFIN profile, otherwise a query receives only a “RIFIN” annotation. Similarly, for the STEVOR HMM profiles, scores between 100 and 145 were considered “likely STEVORs,” and scores ≤ 100 were “unlikely STEVORs.” STRIDE does not report queries that are vastly different to any of the profiles.

## Discussion

To compare sensitivity and specificity between tools, we adjusted the parameters of PFAM and TIGRFAM to match those of STRIDE. STRIDE detected STEVORs in the curated 3D7 reference genome with similar sensitivity to PFAM and TIGRFAM, although sensitivity of STRIDE to detect RIFINs was higher, but this was not statistically significant (*p* = 0.30; χ^2^ = 2.41, DF = 2, Table 2). Specificity to 3D7 sequences was equivalent across all tools. Unlike PFAM and TIGRFAM, STRIDE was not trained using the entirety of RIFINs and STEVORs from the 3D7 repertoire (Fig. 2, Additional file 1: Fig. S4).

**Table 2:** Depicting the sensitivity and specificity analyses of STRIDE compared to PFAM^#^ and TIGRFAM^#^ using 3D7^^^

		RIFIN (185)	Non-RIFIN	Predictive value		STEVOR (43)	Non-STEVOR	Predictive value
STRIDE	Called +	182	0	100%	Called +	38	0	100%
	Called −	3	5363	100%	Called −	5	5505	99.9%
		**98.4%**	100%			**88.3%**	100%	
PFAM	Called +	178	0	99%	Called +	38	0	100%
	Called −	7	5363	99.8%	Called −	5	5505	99.9%
		**96.2%**	99.9%			**88.3%**	100%	
TIGRFAM	Called +	177	0	100%	Called +	38	0	100%
	Called −	8	5363	99.8%	Called −	5	5505	99.9%
		**95.70%**	100%			**88.3%**	100%	

^#^We made comparisons across tools using the same parameters as STRIDE

^^^The curated 3D7 reference genome served as a gold standard. There are a total of 5548 sequences in the *P. falciparum* 3D7 reference, where 182 sequences are annotated as RIFINs and 43 sequences are annotated as STEVORs (includes 27 RIFIN and 10 STEVOR pseudogenes). Unlike PFAM and TIGRFAM, STRIDE was not trained with the entire 3D7 RIFIN and STEVOR repertoire (Fig. 2). The bolded text illustrates the sensitivity of each program; all three tools had 100% specificity

The “validation” dataset spanned a range of variant surface antigen sequence sizes, which included 3888 presumed RIFINs and STEVORs from sequenced clinical isolates and publicly available assemblies (Table 1). STRIDE detected a total of 3540 RIFIN and STEVOR sequences (91.0%), more than the counts for PFAM (2707, 69.6%; p < 0.00001, χ^2^ = 31.30, DF = 1) or for TIGRFAM (3394, 87.3%; p = 0.31716, χ^2^ = 1.00, DF = 1). We also used other *Plasmodium* reference genomes to further test for specificity. STRIDE appropriately detected RIFINs and STEVORs in gorilla- and chimpanzee-infecting parasites (*e.g. P. reichenowi*) but did not register any hits to the genomes of *P*. *vivax*, *berghei*, or *chabaudi*, three species that lack RIFIN and STEVOR orthologs (Table 1).

Using STRIDE, we reevaluated a subset of 320 sequences from PlasmoDB that had received a broad, overlapping “RIFIN/STEVOR family, putative” designation (Additional file 3). These sequences originated from long read-based assemblies of several parasite strains [13]. Among the 312 sequences that met or exceeded identification thresholds, 176 were determined to be RIFIN-As, including 52 with FHEYDER motifs; 80 were RIFIN-Bs; and 56 were STEVORs. Eight sequences did not meet the designated limits of detection for exact classifications. These were mostly truncated copies and thus classified by STRIDE as “RIFIN” or “likely RIFIN.”

We also applied STRIDE to predict the number and classification of RIFINs and STEVORs from 15 unannotated long read-based de novo assemblies of clinical field isolates (Additional file 3) [12]. Initial classification using BLASTp led to mixed results and overlapping annotations. The number of STRIDE-predicted RIFINs and STEVORs from the NF54 de novo assembly mirrored that of 3D7, which was expected given that 3D7 is a clone of the NF54 isolate [14]. STRIDE also consistently identified comparable numbers of RIFINs, STEVORs, and FHEYDER motifs across most clinical samples from diverse geographies. Several “likely RIFIN” sequences from each assembly are encoded by short, truncated contigs in each assembly and could not be precisely classified. There were proportionally greater numbers of sequences found in the Myanmar samples, which are likely polyclonal (Additional file 3).

## Conclusions

We present STRIDE, an HMM-based, command-line program that automates RIFIN and STEVOR prediction, differentiates RIFIN-As from RIFIN-Bs, and identifies the number of sequences with the known pathogenic FHEYDER motif. STRIDE eliminates the need to perform multiple sequence alignments and phylogenetic analyses or to use specialized knowledge of these two protein families to sort RIFINs and STEVORs. STRIDE has better sensitivity to detect RIFINs than other available HMM-based tools and supports adjustable thresholds to customize desired levels of sensitivity and specificity. This HMM-based approach for variant surface antigen classification may be useful for other *Plasmodium* species and organisms with multigene families, such as *Trypanosoma.*

## Supplementary Information

**Additional file 1**. Word document with information on the creation of STRIDE as well as sequences used for generating, training, and validating each HMM profile.

**Additional file 2**. README text file containing information for the user about the STRIDE program, including instructions on its installation and execution and the interpretation of output.

**Additional file 3** List of reclassified PlasmoDB sequences and annotated de novo assemblies using STRIDE. Excel document containing a list of PlasmoDB gene IDs with their original annotations and the suggested classifications based on STRIDE, in addition to the predicted number of RIFINs and STEVORs from 15 de novo assemblies with their respective accession numbers.

## Data Availability

The datasets with their respective accession numbers supporting the conclusions of this article are listed in Additional file 3. Other datasets used and/or analyzed in this current study are available from the corresponding author upon request. *Project Name:* STRIDE (STevor RIfin iDEntifer) *Project Home Page:* https://github.com/albert-zhou-umb/STRIDE.git *Operating system:* Platform-independent *Programming Language:* Command-line application written in Bash *Other Requirements:* HMMERv3.3 and Perl v5.24 *License:* GNU GPL *Any Restrictions to Use by Non-Academics*: None.

## Abbreviations

3D7: Reference strain and lab variant of *P. falciparum*
BLASTn/p: Basic local alignment search tool (nucleotide/protein)
FHEYDER: A seven amino acid motif of RIFIN-As that bind to B- and NK-cells
HMM(ER): Hidden Markov model
PFAM: Database of protein families to support genome annotation
RIF/RIFIN: Repetitive interspersed family of polypeptides protein
RSpred: RIFIN/STEVOR predictor
SC: Semi-conserved domain
STEV/STEVOR: Sub-telomeric variable open reading frame protein
STRIDE: STEVOR and RIFIN Identifier
TIGRFAM: Database of protein families to support genome annotation
V1/V2: Variable domain 1/variable domain 2

## References

[1] Gardner MJ, Hall N, Fung E, White O, Berriman M, Hyman RW (2002). Genome sequence of the human malaria parasite *Plasmodium falciparum*. Nature.

[2] Goel S, Palmkvist M, Moll K, Joannin N, Lara P, Akhouri R (2015). RIFINs are adhesins implicated in severe *Plasmodium falciparum* malaria. Nat Med.

[3] Travassos MA, Niangaly A, Bailey JA, Ouattara A, Coulibaly D, Lyke KE (2018). Children with cerebral malaria or severe malarial anaemia lack immunity to distinct variant surface antigen subsets. Sci Rep.

[4] Joannin N, Kallberg Y, Wahlgren M, Persson B (2011). RSpred, a set of hidden Markov models to detect and classify the RIFIN and STEVOR proteins of *Plasmodium falciparum*. BMC Genomics.

[5] Petter M, Haeggström M, Khattab A, Fernandez V, Klinkert M-Q, Wahlgren M (2007). Variant proteins of the *Plasmodium falciparum* RIFIN family show distinct subcellular localization and developmental expression patterns. Mol Biochem Parasitol.

[6] Saito F, Hirayasu K, Satoh T, Wang CW, Lusingu J, Arimori T (2017). Immune evasion of *Plasmodium falciparum* by RIFIN via inhibitory receptors. Nature.

[7] Zhou AE, Berry AA, Bailey JA, Pike A, Dara A, Agrawal S (2019). Antibodies to peptides in semiconserved domains of RIFINs and STEVORs correlate with malaria exposure. mSphere..

[8] Altschul SF, Gish W, Miller W, Myers EW, Lipman DJ (1990). Basic local alignment search tool. J Mol Biol.

[9] Eddy SR. HMMER user’s guide. 2018:221.

[10] Haft DH, Selengut JD, Richter RA, Harkins D, Basu MK, Beck E (2013). TIGRFAMs and genome properties in 2013. Nucleic Acids Res.

[11] Finn RD, Attwood TK, Babbitt PC, Bateman A, Bork P, Bridge AJ (2017). InterPro in 2017-beyond protein family and domain annotations. Nucleic Acids Res.

[12] Moser KA, Drábek EF, Dwivedi A, Stucke EM, Crabtree J, Dara A (2020). Strains used in whole organism *Plasmodium falciparum* vaccine trials differ in genome structure, sequence, and immunogenic potential. Genome Med.

[13] Otto TD, Böhme U, Sanders M, Reid A, Bruske EI, Duffy CW (2018). Long read assemblies of geographically dispersed *Plasmodium falciparum* isolates reveal highly structured subtelomeres. Wellcome Open Res.

[14] Walliker D, Quakyi IA, Wellems TE, McCutchan TF, Szarfman A, London WT (1987). Genetic analysis of the human malaria parasite *Plasmodium falciparum*. Science.

