# Supplementary material for: STRIDE: a command-line HMM-based identifier and sub-classifier of Plasmodium falciparum RIFIN and STEVOR variant surface antigen families
Source: BMC Bioinformatics. 2022 Jan 6;23:15. doi: 10.1186/s12859-021-04515-8 (PMC8733436; doi:10.1186/s12859-021-04515-8)
Supplement: Supplementary file 1 — Additional file 1. Word document with information on the creation of STRIDE as well as sequences used for generating, training, and validating each HMM profile. [file 12859_2021_4515_MOESM1_ESM.docx]

**STRIDE: a command-line HMM-based identifier and sub-classifier of *Plasmodium falciparum* RIFINs and STEVORs**

Albert E. Zhou, Zalak V. Shah, Katie Bradwell, James B. Munro, Andrea A. Berry, David Serre, Shannon Takala-Harrison, Timothy D. O’Connor, Joana C. Silva, and Mark A. Travassos

**Supplemental Information**

**Supplemental Figure S1. Flowchart of the general process used to generate RIFIN-A, RIFIN-B, and STEVOR HMM profiles.**

**Supplemental Figure S2. Flowchart of the sequences used for training and validating the RIFIN-A, RIFIN-B, and STEVOR profiles.**

**Supplemental Figure S3. Relationships of HMM Scores using reference genome 3D7 positive controls.** A set of positive control sequences from 3D7 illustrates profile thresholds, as shown in the Circos plot for each group. The circumference represents the whole sequence score, and the radius represents the HMM domain score.


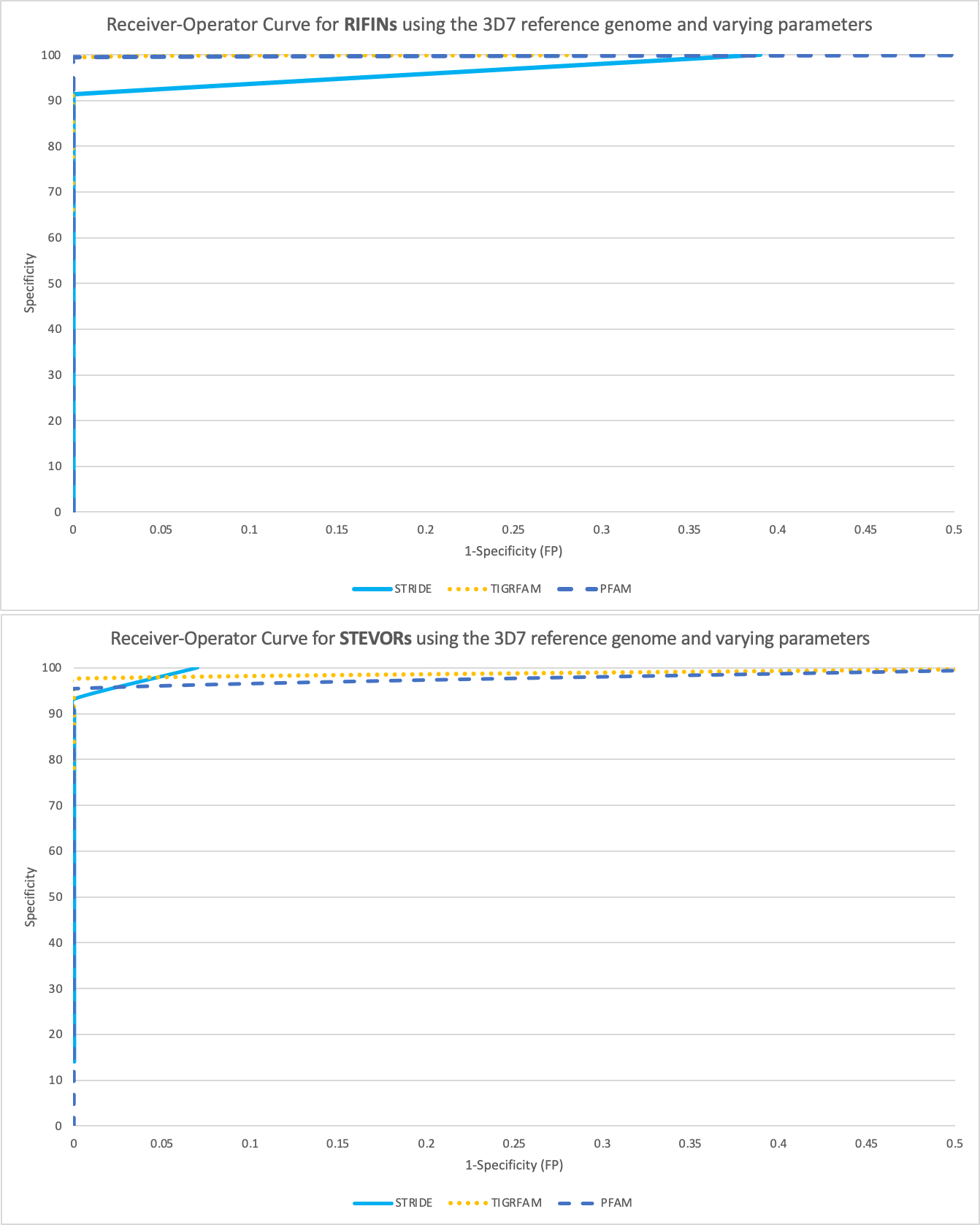


**Supplemental Figure S4: Receiver-Operator Curves for RIFINs and STEVORs comparing STRIDE to PFAM and TIGRFAM using the 3D7 reference genome.** FP is “false positive.”
